# Development of a Multicomponent Adsorption Isotherm Equation and Its Validation by Modeling

**DOI:** 10.1021/acs.langmuir.3c02496

**Published:** 2023-11-24

**Authors:** Amrutha Acharya, Gautham Jeppu, Chikmagalur Raju Girish, Balakrishna Prabhu

**Affiliations:** Department of Chemical Engineering, Manipal Institute of Technology (MIT), Manipal Academy of Higher Education (MAHE), Manipal 576104, Karnataka, India

## Abstract

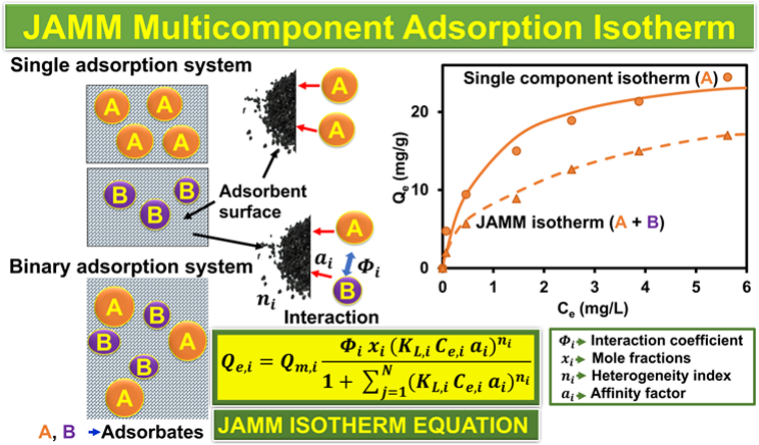

Researchers have made significant efforts over the past few decades to understand adsorption by developing various simple adsorption isotherm models. However, though many contaminants usually occur as multicomponent mixtures in nature, multicomponent adsorption isotherms have received limited attention and remain an area of inadequate research. We have presented here in a new multicomponent adsorption isotherm model, named the Jeppu Amrutha Manipal Multicomponent (JAMM) isotherm, that can alleviate this problem. We first developed the JAMM multicomponent isotherm using our experimental data sets of arsenic and fluoride competitive adsorption on activated carbon. We then tested the JAMM multicomponent isotherm for a case study of cadmium and zinc competitive adsorption. Next, we further assessed the JAMM isotherm using another competitive adsorption case study of copper and chromium. Through extensive validation studies and error analysis, the JAMM isotherm was able to demonstrate its efficacy in predicting the adsorption behavior in several multicomponent adsorption systems accurately. The main advantage of JAMM isotherm over other multicomponent isotherms is that it utilizes and leverages the single-component adsorption parameters to simulate multicomponent isotherms. The proposed JAMM analytical isotherm model furthermore incorporates the interaction between the components, a mole fraction parameter, and a heterogeneity index, providing a more comprehensive modeling framework for multicomponent adsorption. The mole fraction term was introduced for the distribution of adsorption sites based on the relative number of molecules of each component. An additional term for interaction coefficient was introduced for the representation of interactions. During the validation of JAMM with three experimental case studies with negligible, small, and high competition systems of adsorbates, impressive predictions were exhibited, with the average normalized absolute percentage error as 6.05% and average *R*^2^ as 0.86, highlighting the model’s robustness, versatility, and reliability. We propose that the new JAMM isotherm modeling framework might profoundly help in chemical engineering, environmental engineering, and materials science applications by providing a potent tool for analyzing and predicting multicomponent adsorption systems.

## Introduction

The rapid increase in water pollution from industrialization, mining activities, and other sources has led to the discharge of heavy metals, organic compounds, and various inorganic pollutants into drinking water sources. Traditionally, single-component adsorption studies have addressed the removal of contaminants, such as heavy metals and organic compounds. However, the truth of real-world water contamination problems is far more complex, often harboring multiple pollutants that interact strongly, influencing the adsorption process. The multicomponent adsorption studies have gained relevance for their advantage in simultaneously removing various pollutants in water sources.^1,2^ However, multicomponent adsorption isotherm models have been relatively unexplored and remain an area of restricted research due to its complexity.^3,4^ As concerns about water pollution escalate, researchers are increasingly exploring innovative approaches to tackle the challenge of the simultaneous removal of multiple contaminants from aqueous environments.^9,10^ Development of adequate multicomponent models is crucial to navigating complex multicomponent adsorption interactions and global water treatment issues. By conducting research on adsorption, many researchers have strived to alleviate the impact of water pollution to ensure the preservation of precious water resources for a sustainable and progressing future.^5,6^ This cumulative effort has been driven by the desire to unravel the intricacies of adsorption phenomena in diversified environments and industrial contexts.

In pursuit of enhanced adsorption efficiency, researchers have delved into the complex interaction between contaminants and adsorbents, leveraging novel materials and employing analytical modeling frameworks.^9,10^ Their efforts have led to the establishment and refining of various equilibrium isotherm models and provided relevant tools to interpret the behavior of solutes on solid surfaces at specific temperatures. Preceding researchers have made consequential attempts in addressing the challenges of multicomponent adsorption by developing equilibrium isotherm models.^7^ These mathematical formulations serve as beneficial tools for comprehending the behavior of different pollutants in a system. These techniques help researchers design superior adsorbents, optimize adsorption processes, and derive more competent and viable solutions for various industrial and environmental applications.^8^

Generating mathematical equations that accurately depict the data collected during adsorption assessments is imperative for modeling and simulating adsorption isotherms. Experimental data acquired through adsorption investigations was used to set the parameters of isotherm models.^9−11^ The gathered data were fitted to the corresponding model equations using various regression techniques, including the sum of square error, root-mean-square error, chi-square error, and average percentage error approaches to attain the best-fit parameters.^12,13^

Several notable multicomponent models have emerged through rigorous investigation, offering unique insights into the intricate interactions between pollutants.^14,15^ At the same time, many existing multicomponent isotherms are based on assumptions, and have their advantages, and limitations. Earlier multicomponent isotherms like extended Langmuir (EL), extended Langmuir–Freundlich, modified competitive Langmuir (MCL), modified competitive Redlich–Petersons, Sheindorf–Rebuhn–Sheintuch,^16,17^ extended SIPS, modified Jain–Snoeyink, steric mass action,^1^ and Freundlich–Langmuir–Jovanovic isotherms, etc. have broad applications. These models have been used for simulating complex adsorption phenomena and predicting adsorption behavior. Despite their broad applications, the existing isotherm models have certain limitations, as well.

Myers and Prausnitz developed the ideal adsorbed solution theory (IAST), a theoretical framework used to predict multicomponent mixtures and provide individual components’ behavior in a mixture. It assumes that the mixture behaves as an ideal solution on the adsorbent surface, which means there are no interactions between the adsorbed species. It relies on the availability of single-component isotherms for each component in the mixture. However, IAST considers only gaseous mixtures, which are only practically applied for some of the aqueous adsorption systems, which was a rather significant setback.^18^ It assumes uniform adsorption sites of the adsorbent, but in reality, some surfaces can be heterogeneous. Based on equilibrium assumptions, the model was more accurate for low concentrations than high concentrations, which might not be suitable for several applications.^19^ No other isotherms except IAST have included the mole fraction. The IAST model is, however, not suited for the simulation of heavy metal adsorption in aqueous systems, and is more complex and tiresome for application.

Similarly, the major drawback of the extended Langmuir isotherm model is that the surfaces of adsorbents are assumed to be homogeneous and monolayered. However, many adsorptive surfaces may have heterogeneous surfaces with various affinities toward different pollutants.^20,21^ Also, the binding sites are independent, and the interaction phenomena between the pollutants and the adsorbent surface are ignored in this EL model. Similarly, the extended Freundlich (EF) isotherm is modified with an exponential distribution, and a number of constant parameters has to be optimized simultaneously, leading to inaccuracies. Also, the EF isotherm assumes the adsorbent surface as heterogeneous, but neglects the interaction of adsorbate–adsorbate and adsorbate–adsorbent in the adsorption process.^8^

Likewise, although the modified competitive Langmuir (MCL) isotherm includes the interaction coefficient, it ignores the heterogeneity index and mole fractions. The MCL isotherm behaves as the EL isotherm when the interaction coefficient is unity, and the maximum uptake of the multicomponent is predicted after each fitting for every dataset.^1,8,22^ Markham and Benton’s approach has expanded EL to a multicomponent adsorption model with interaction parameters but assuming monolayer adsorption and mole fractions. The multicomponent capacity factor was considered in the *p*-factor model developed by Mckay and Al Duri, where *p* represents all competitive effects together.^23^

Nevertheless, the conventional multicomponent adsorption models have generally overlooked crucial competitive factors, such as interaction coefficient, adsorbate–adsorbent affinity, surface heterogeneity, and mole fractions, significantly impacting the competitive adsorption isotherm’s predictive ability. An additional constraint in the existing multicomponent isotherms is that a limited number of pollutants have to be considered and typically can handle no more than three or four pollutants, exhibiting complex challenges.^8^ Additionally, conducting experiments for the simultaneous removal of pollutants is tedious, leading to high experimental errors and inaccuracies in the model prediction. Hence, multicomponent experimental data are limited and scarce for developing and testing the performance of multicomponent isotherm models. Furthermore, testing, refining and validating the parameters of the developed multicomponent adsorption models is a laborious process.^24,25^

**Figure 1:**
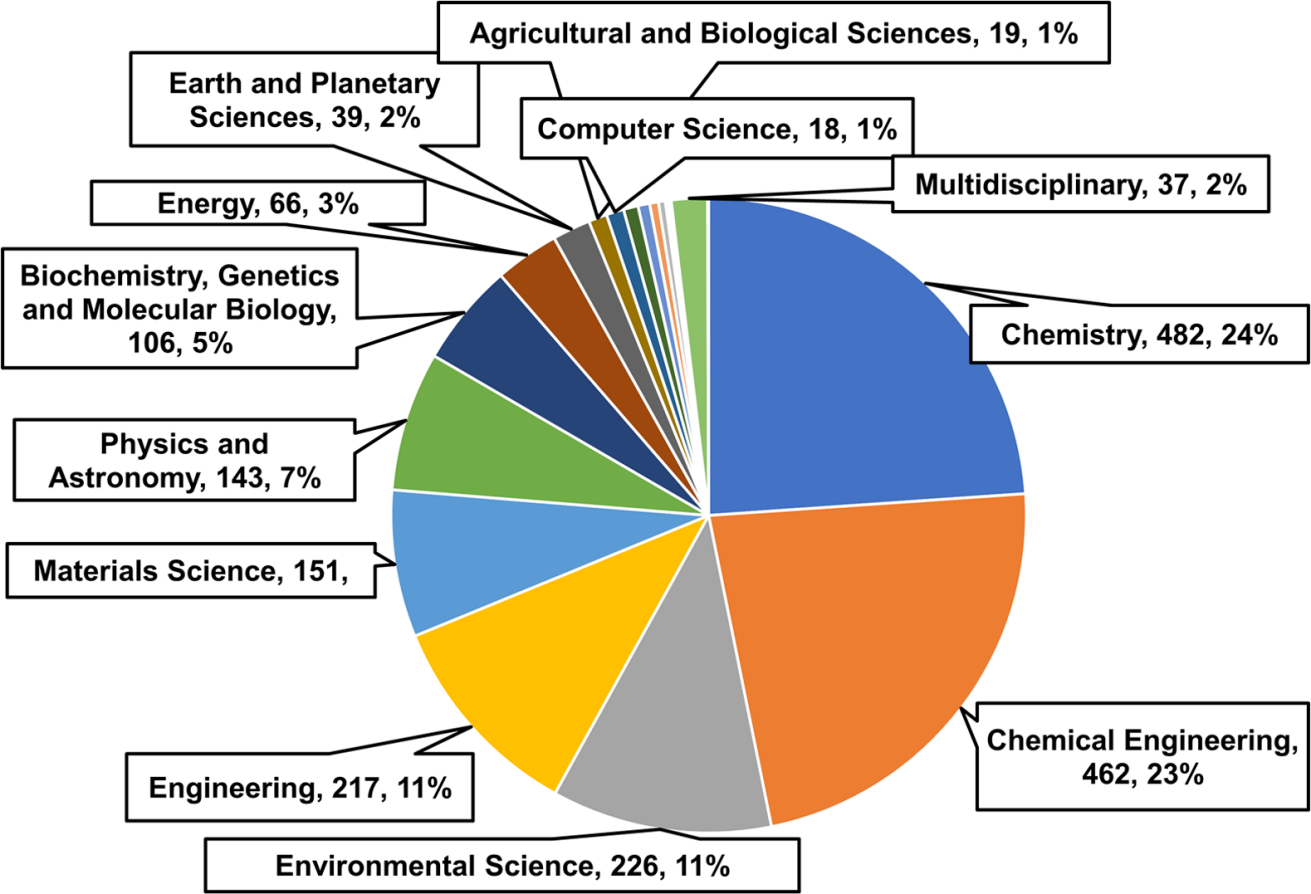
Publications in various subject areas, as per Scopus.

**Figure 2:**
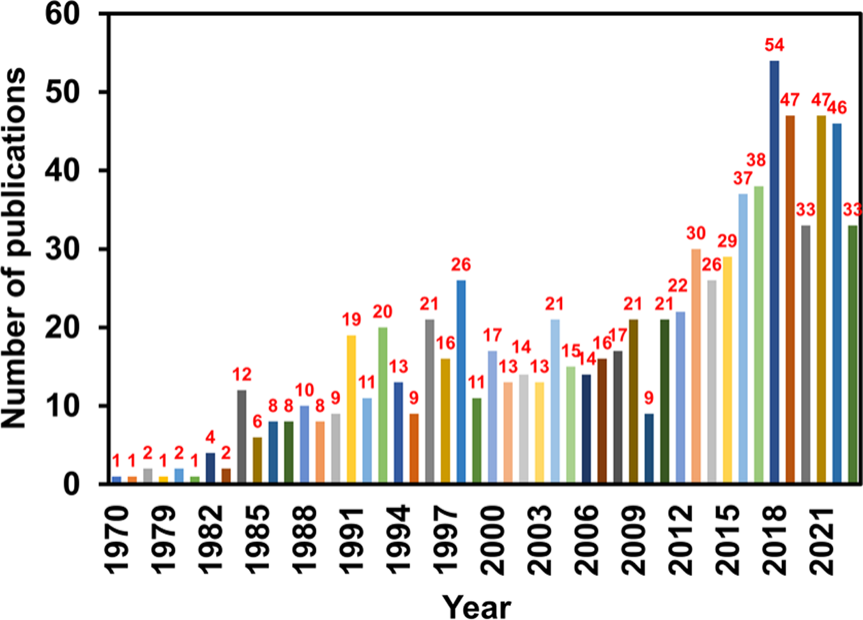
Number of publications on multicomponent adsorption over the years, as per Scopus.

To survey the importance of multicomponent adsorption isotherms, several scientific publications from 1970 to 2023 were retrieved from the Scopus search engine, which is a powerful research tool. The search query keywords were “multicomponent adsorption isotherm models”, “binary adsorption isotherm models”, and “competitive adsorption isotherm models”. A total of approximately 854 documents were published on these topics. The gathered information is visually summarized in two distinct graphs given below. In Figure 2, a bar chart illustrates the number of publications over a specified time frame. The pie chart offers insights into diverse research disciplines in a particular field, as shown in Figure 1, determining the study’s scope. The research in multicomponent adsorption in disciplines such as chemistry, chemical engineering, material science, genetics, and molecular biology has remarkably progressed. The number of publications in this area increased linearly over the period.

While there were comparatively few studies on multicomponent adsorption models in the previous three decades, the rate of research output has surged with an average of 45 research articles per year across various domains during 2018–2023. The significant fields showing interest in this area are chemistry (23%), chemical engineering (24%), environmental science (11%), and several others. Therefore, this demonstrates that multicomponent adsorption has significant contributions and relevance in driving recent advancements in many fields. The number of publications has increased constantly, showing growing research interest in multicomponent adsorption.

The objective of this research is to introduce a new analytical isotherm model to simulate competitive adsorption equilibrium systems effectively. A distinct advantage of a new Jeppu Amrutha Manipal multicomponent (JAMM) isotherm model is its ability to account for interaction phenomena and mole fraction, setting it apart from the existing isotherm models. Its computational application was demonstrated by accurately modeling diverse sorption case studies with a very low error, reaffirming its potential for practical and real-world applications. The JAMM isotherm model derived in this current study was thus demonstrated to be an analytical, computational, and systematic approach for multicomponent systems that considers significant parameters, such as an interaction coefficient between pollutants and adsorbents, mole fractions, affinity factor, and heterogeneity index, thus providing a more comprehensive representation of adsorption mechanisms neglected by other multicomponent isotherms.

## Methods

### New Analytical Approach—JAMM Isotherm Adsorption Model

In this section, we develop the JAMM isotherm model. The analytical framework, statistical approaches, experimental procedures used to derive the model, and statistical methods used to describe the performance of the developed model are also explained. In our earlier studies,^7^ the Langmuir isotherm was altered to a novel model, the modified Langmuir Freundlich isotherm (MLF), a pH-dependent adsorption model. We considered different data sets for arsenic adsorption in a single-component system employing pure goethite and goethite-coated sand.^26^ The experimental data sets were simulated using surface complexation modeling, and the modified model had an improved ability to predict pH-dependent data sets.^7,27^ This present work focuses on developing a novel analytical multicomponent isotherm model by expanding the widely used Langmuir isotherm model in its extended form. The factors influencing the adsorption mechanism and competitive parameters of simultaneous systems are also discussed below.

### Parameters and Mechanisms Influencing Competitive Adsorption

A notable problem in surface science is the lack of analytical solutions, which necessitates derivation of precise adsorption equilibrium expressions. At the same time, some modern statistical mechanisms provide a framework to determine the behavior of many-particle and surface systems.^28−30^ The key to theoretical adsorption studies is a precise formulation of the isotherm, where equilibrium adsorption depends on many parameters associated with the nature of the adsorbate and adsorbent.^30,31^

The distinctiveness of this JAMM isotherm (eq 14) lies in the fact that four extra parameters are added to modify the EL isotherm model (eq 2) to attain a more appropriate competitive fit and reduce errors. The four parameters selected are the heterogeneity index, affinity factor, mole fraction, and interaction coefficient, which are explained below.

### Heterogeneity Index

The heterogeneity index is a fundamental parameter incorporated to depict the intricate interplay between the adsorbate and adsorbent. It was recognized that actual surfaces are not homogeneous to a greater or lesser extent; hence, the adsorbent heterogeneity is entailed in advanced adsorption research.

Nevertheless, many researchers have contributed to adsorption theory on heterogeneous surfaces and interpreted experimental data. Competitive adsorption isotherms like EF, IAST, MLF, Langmuir Freundlich hybrid, simplified ideal adsorbed solution, and improved simplified ideal adsorbed solution models have introduced the term heterogeneity index. Adsorbing multiple pollutants in a simultaneous system requires a heterogeneity index to describe the surface of the adsorbent material. Therefore, the heterogeneity index was incorporated in the new JAMM framework and expressed as *n*_*i*_ and *n*_*j*_ for the components *i* and *j*. The heterogeneity index ranges from 0 to 1. For a homogeneous material, the value of *n* is 1; if it is below 1, it is heterogeneous. When the *n*_*i*_ and *n*_*j*_ values approach 1, the equation reduces to the extended Langmuir isotherm model.

### Affinity Factor

Affinity can be defined as the attraction energy of pollutants toward the sorbent surface. Factors impacting the energy distribution of active adsorbent sites can be attributed to the various surface properties, defects, impurities, vacancies, and chemical contaminants. As a result, the adsorption energies of all the active sites exhibit variations.^32,33^ A field of active sites with the potential to attract pollutant molecules depends on the active sites’ functions and spatial positions. Anionic pollutants tend to attract more strongly to the positively charged functional groups of adsorbents, while cationic sorbates have a higher affinity toward negatively charged sorbent surfaces. Consequently, the adsorption is influenced by active sites, contributing to its energy value to attract and retain multiple components of interest. Researchers have introduced affinity parameters in previous multicomponent isotherms, such as the MLF isotherm and Levan–Vermeulen models. Hence, it was imperative to consider the affinity parameters to account for variables and effectively manage pollutants.^34^ Therefore, the affinity factor was considered and denoted as *a*_*i*_ and *a*_*j*_ for components *i* and *j* in the new analytical isotherm. The affinity factor characterizes the adsorption tendencies. It directly correlates to the affinity between the adsorbates and adsorbents of the system.

### Interaction Coefficient

Various contaminants in the solution introduce a complex interplay affecting ion diffusion and the driving forces to reach the active sites. Depending on the specific interactions, this complexity can lead to antagonistic or synergistic mechanisms.^35,36^ Several critical factors are considered to understand model mechanisms, including molecular size, ionic radii, hydrated radii, and diffusivities, which influence the competition among different species within the solution. The interaction between the adsorbent surface and the mobility of adsorbates toward active sites is addressed by this model. These interactions between adsorbents impact adsorption systems, often resulting in intricate dependencies.^37^ Increasing the concentration of one component may alter another component’s adsorption behavior, leading to an equilibrium. In many scenarios, the number of ions adsorbed in a single-component system exceeded that in a simultaneous multicomponent system, attributed to size-exclusive effects, particularly when larger and smaller molecules coexist within the same system.^38,39^ Furthermore, in simultaneous removal scenarios, the interaction of different components toward active sites can vary, and the adsorption of one component may cause steric hindrance on the other. These size-based restrictions are significant when molecules of different sizes compete for the same adsorption active sites.^40,41^

For multicomponent systems, the competition among different adsorbates inhabiting the pores of the adsorbent is closely tied to factors such as the pore size, molecular size, and interactions occurring within these confined pores. Langmuir extension first approximation, Sheindorf Rebuhn Sheintuch, empirical bisolute extension of Freundlich, MCL, and modified competitive Redlich Peterson isotherm models have interaction coefficients as the parameter. Accordingly, the interaction coefficient was employed in our new computational multicomponent isotherm. The interaction coefficient can be represented as Φ_*i*_ and Φ_*j*_ for components *i* and *j*. This coefficient predicts nuance among different adsorbates and their interactions within the adsorption process, offering a more comprehensive framework for understanding and optimizing adsorption systems.^42,43^ Consequently, the interaction coefficient was also included in JAMM to identify the behavior of one component in the presence of another.

### Mole Fraction

The other parameter that represents the fraction of moles in the multicomponent solution is the mole fraction, which is used to quantify the fraction of the number of solute molecules in the substances in the mixture. Mole fractions can also be defined as the ratio of number of moles of a component to the total number of moles in the mixture. The sum of mole fractions of all components in a multicomponent solution is always 1, and the numerical range of mole fractions is between 0 and 1. Mole fractions are essential in analyzing chemical reactions, partial pressures, and solubility. A significant change in the number of contaminants increases the system’s complexity due to increased molecular and ionic interactions. Molecules interact with one another, where mole fractions quantify the relative molecular species within the mixture, showing the binding sites, space, and coexistence. The IAST and IAST Freundlich models have used mole fraction terms. Accordingly, a mole fraction term was introduced in our new JAMM isotherm model. The molecular parameters varying the adsorption properties are given in Table 1.

**Table 1:** Correlation between Atomic Level Parameters and the Adsorption Process

sl. no	atomic parameters	influence on adsorption	refs
1	ionic radius	it influences the surface chemistry and the ion-exchange. The larger the ionic radii, the more challenging it is to fit into the adsorbent’s pore size. However, smaller ions with smaller ionic radii can easily access the active sites of the adsorbent, influencing more adsorption. The ion selectivity for suitable adsorbent pore size can be exploited preferentially. Additionally, smaller ions with high charges may favor the adsorption processes. The smaller the size, the higher is the tendency to interact with the bulk active sites, enhancing the adsorption as well. In multi-ion systems, smaller ions are more competitive in occupying the available adsorption sites	(47−48,49)
2	hydrated radius	the hydrated radii of ions show the effective size of ions in the solution. Larger hydrated ions have a larger effective size. The adsorption sites preferably adsorb the ions with smaller hydrated radii. As a result, smaller hydrated ions may be favored for adsorption due to their small size. The hydration thickness around the larger ions may retain more water molecules, which can influence the interaction with the surface of the adsorbents; the smaller the hydration thickness, the more is the interaction and the more is the adsorption	
3	electronegativity	a measurement of the force with which a species tends to attract the electrons. It can affect the adsorbent surface and adsorbate bonding, leading to polar interactions. The higher electronegativity of molecules results in the redistribution of electron density, which in turn affects the adsorption capacity. Functional groups on various adsorbent surfaces may create sites with different affinities for different adsorbate molecules, leading to selective adsorption in the competitive adsorption system. The adsorbents with electronegative functional groups may attract anions, while electropositive surface functional groups attract the anions	
4	diffusion coefficient	the adsorption process is inversely proportional to the diffusion, i.e., the rate of adsorption increases when the diffusion coefficient typically decreases. The higher the diffusion coefficient, the faster the adsorbate molecules reach the active adsorbent sites. A higher diffusion coefficient, shorter time for adsorption equilibrium, and lower diffusion coefficient can lead to extended time to reach equilibrium	

Raoult’s law is uses mole fractions to calculate the vapor pressure of an ideal solution depending on the mole fractions of its components. Raoult’s law assumes that the binary solution displays a behavior identical to an ideal solution. It also postulates that the components’ interactions are similar to the molecules’, which implies that there would be no interaction among the components.^11,25,44^ In conclusion, the component with a higher mole fraction corresponds to a higher tendency to adsorb as more moles are available for interaction. It was also assumed that the adsorption mole fraction distribution is similar to Raoult’s law.^45^ Consequently, the mole fractions are included in JAMM, as they provide a valuable fundamental principle of the composition of mixtures.

Mathematically, mole fractions can be expressed as below for a mixture of two components in the solution. For a binary mixture, the two components are presumed to be *i* and *j*. The mole fractions can be denoted as *x*_*i*_ and *x*_*j*_ for components *i* and *j*. The mole fraction was formulated as the ratio of the number of moles of component *i* to the sum of number of moles of components *i* and *j*. For a new multicomponent model, JAMM (eq 14) leveraged the EL isotherm/nonmodified Langmuir isotherm model (eq 2) as the basic model.^46^ Furthermore, Table 1 explains the other molecular level parameters and how they affect adsorption.

The new approach, the JAMM isotherm model, was implemented to partly refine some of the existing multicomponent isotherm models. The JAMM isotherm represents a reliable and flexible framework to explain adsorption with a consistent use of parameters. The developed model has its advantages and limitations. The notable advantages were that the proposed equation was straightforward to use; it allows single-component values to be used to predict multicomponent adsorption, showing its versatility, and the adsorption equation follows distribution according to Raoult’s law, providing a theoretical basis. The drawbacks are that it needs nonlinear solving of equations to determine constants such as interaction coefficients and affinity factor; the inclusion of heterogeneity index increases the complexity, and this analytical approach is more suitable for Langmuir type of isotherms. The development of the new multicomponent isotherm incorporating the mentioned parameters was done as described below starting from the Langmuir isotherm model.

### Langmuir Isotherm Model

The Langmuir isotherm model (eq 1) is the fundamental framework for simulating a single solute system in this investigation. The functionality of the Langmuir isotherm lies in the assumption that adsorbent sites possess a uniform energy to attract adsorbates. This model postulates the formation of a monolayer on a homogeneous surface, with no interactions occurring between the adsorbates. The Langmuir isotherm considers the interactions between adsorbates and adsorbents as negligible or nonexistent.^50^

The Langmuir isotherm model is given by
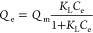
1where *C*_e_ is the adsorbate concentration at equilibrium (mg/L), *Q*_e_ is the equilibrium adsorption capacity of adsorbents (mg/g), *Q*_m_ is the maximum adsorption capacity (mg/g), *K*_L_ is the Langmuir constant expressed as L/mg.

### EL Isotherm Model

An extension of Langmuir isotherm model is given by the extended Langmuir (EL) model. Within the isotherm model (eq 2), uniform active sites on the adsorbent are presumed to possess equal energy, facilitating the adherence of pollutant molecules through noninteracting actions, nevertheless, as the isotherm parameters’ values may significantly vary when it is applied to other adsorption systems.^16^

The EL isotherm/nonmodified Langmuir isotherm model (EL) is given by
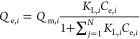
2where *Q*_e,*i*_ and *Q*_e,*j*_ are the equilibrium adsorption capacities for components *i* and *j* (mg/g), *Q*_m,*i*_ and *Q*_m,*j*_ are the maximum adsorption capacities for components *i* and *j* (mg/g), *K*_L,*i*_ and *K*_L,*j*_ are the Langmuir constants for components *i* and *j* (L/mg), and *C*_e,*i*_ and *C*_e,*j*_ are the equilibrium concentrations for components *i* and *j* (mg/L).

### Development and Derivation of the Proposed JAMM Isotherm Model

In the mathematically generalized development and derivation of the proposed isotherm, the adsorption rate is directly proportional to the number of vacant sites (*N*_A_), the bulk fluid concentration (*C*_s,_ mg/L), the rate constant (*k*), and the fractions of vacant active sites (θ_v_). Thus, the equation becomes
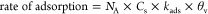
3
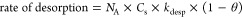
4where θ is the fractional occupancy of the active sites of adsorbents. On equating eqs 3 and 4 and considering the equilibrium between the adsorbate and adsorbent, the rate of adsorption is equal to the rate of desorption
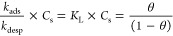

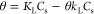

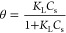
5where  (L/mg); the bonding energy for different types of bonds is measured between the molecules; and at equilibrium, adsorbate loading the adsorbent, i.e., . Thus, on simplification, the equation reduces to the Langmuir isotherm
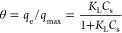
6

Hence, the Langmuir isotherm forms for a single-component adsorption.

Similarly, for the binary system, the adsorption rate is directly proportional to the number of vacant sites (*N*_A_), the bulk fluid concentration of component “*i*” (*C*_s_, mg/L), the rate constant (*k*_*i*_), and the fractions of vacant active sites (θ_v_). Thus, the equation becomes
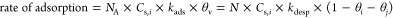
7
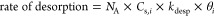
8

At equilibrium, the rate of adsorption equals to rate of desorption, by the solving of eqs 7 and 8 between the adsorbate and adsorbent, with the rate of adsorption equals to the rate of desorption for the components “*i*” and “*j*” gives
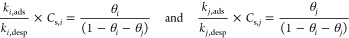

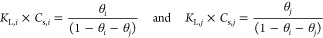


Therefore, for binary systems, on adding the “*i*” and “*j*” components
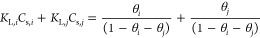


Adding 1 on both sides, we get
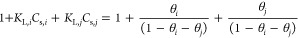

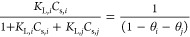

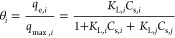
9
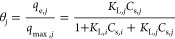
10

Hence, an EL isotherm model is reached by combining the last relationship with the balanced condition. For multicomponent adsorption, the EL isotherm model was added with the parameter heterogeneity index expressed as *n*_*i*_ and *n*_*j*_ for components *i* and *j*, and the equation was modified as
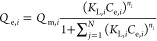
11

The mole fraction can indicate the dependency of the behavioral property of mixtures. The mole fraction of the single component system can be represented as *x*_*i*_ = 1, and *Q*_m_ was corrected as (*Q*_m*i*_ × *x*_*i*_) for multicomponent adsorption systems. The mole fraction is defined as the ratio of the number of moles of the “*i*” component (*M*_*i*_) to the sum of moles of “*i*” and “*j*” components and vice versa. Hence,  and . Therefore, inserting mole fraction in (eq 11), the equation becomes
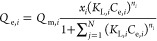
12

Further modifications to the eq 12, factoring the interactions between the molecules and the adsorbent surface, will lead to representation of the competition for multisites. By including the interaction between the adsorbates in the multicomponent phase, the interaction coefficient, with *Q*_m_ can be expressed as (*Q*_m*,i*_ × *x*_*i*_ × Φ_*i*_). This expression (eq 12) is thus modified and thus results in the below equation
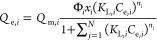
13

Further, while we consider the interactions, it is essential to recognize that some molecules may exhibit a heightened attraction or affinity, increasing their presence at the adsorbent surface’s bulk phase, and could result in steric hindrance for other components. To adjust the affinity changes in the multicomponent phase, an affinity factor “*a*_*i*_” was introduced with *K*_L,*i*_, represented as (*K*_L,*i*_ × *a*_*i*_). Thus, *K*_L,*i*_ was replaced by (*K*_L,*i*_ × *a*_*i*_). Thereafter, (eq 13) leads to the new computational equation called as the JAMM isotherm model. Subsequently, the final JAMM equation is given by eq 14 below
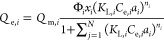
14where, *Q*_e,*i*_ is the equilibrium adsorption capacity for component *i* (mg/g), *Q*_m,*i*_ is the maximum adsorption capacity for component *i* (mg/g), *K*_L,*i*_ is the Langmuir constant for component *i* (L/mg), *C*_e,*i*_ is the equilibrium concentration for component *i* (mg/L), *a*_*i*_ is the affinity factor for component *i* (constant), Φ_*i*_ is the interaction coefficient for component *i* (constant), *x*_*i*_ is the mole fraction for component *i*, *n*_*i*_ is the index of heterogeneity for component *i* (constant), and *N* is the number of components in the adsorption system.

### Validation of the JAMM Model

We conducted experiments and compiled data sets from previous literature studies for analytical modeling. Several equilibrium experimental data sets of single and multicomponent data sets are extracted from literature. We employed the JAMM model to simulate the adsorption parameters and comprehensively describe the adsorption curves. The nature of the curve is predetermined by fitting the model-predicted data sets and experimental values. The optimal model fitting was successfully executed by utilizing the Solver algorithm of Microsoft Excel, i.e., Microsoft Excel Solver. The Excel Solver assists in reducing the sum of square errors and average percentage errors. The convergence of errors accomplished the evaluation of the parameter values.

The values of the maximum adsorption capacity, *Q*_m_ (mg/g), and the Langmuir constant, *K*_L_ (L/mg), are extracted from the single-component Langmuir isotherm model. Those values of *K*_L_ and *Q*_m_ are further deployed in the multicomponent isotherm JAMM model. JAMM (eq 14) was applied to fit all the competitive isotherm data sets simultaneously by reducing the sum of square errors. The error values were evaluated using *R*^2^, MAPE, chi-square, and NAPE, explained in subsequent sections. The coefficient of determination, *R*^2^ values fitting greater than 0.9, indicates the goodness of fit, and lower errors show a better model performance.

**Figure 3:**
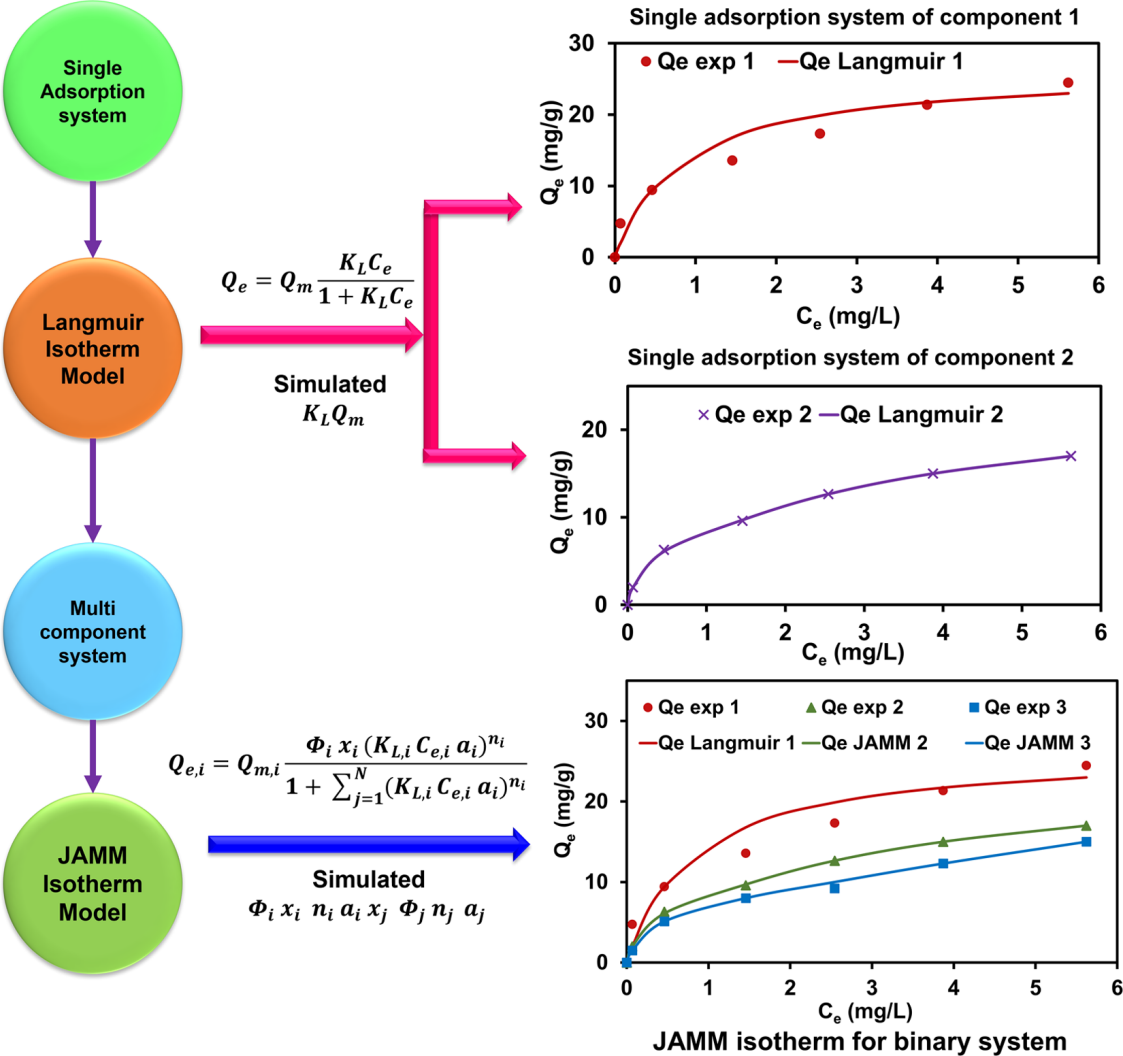
Flowchart of the JAMM isotherm modeling framework.

Numerous isotherm models exist to analyze the simultaneous removal of various pollutants. These isotherms delineate and characterize the sorptive behaviors and provide comprehensive insights into the adsorption system’s overall behavior.^51,52^ The efficacy of the newly developed computational isotherm model must therefore be substantiated with a wide array of multicomponent systems. The modeling of some case studies was conducted using the new analytical framework JAMM (eq 14) for the equilibrated experimental data sets. The validation ascertains the model accuracy assessment, demonstrating the performance, accuracy, and reliability of isotherm models in any adsorption scenario. A validated modeling approach describes the generalized method to predict a diverse range of adsorption systems, irrespective of specific experimental conditions, adsorbates, and adsorbents. Thus an appropriate modeling approach for a particular adsorption system has to be selected, and its performance validated for a given application.^53^ The step-by-step modeling methodology for developing and validating JAMM isotherm is shown in Figure 3.

### Statistical Error Analysis Methods

The isotherm models are acceptable only when the errors between the experimental and predicted values are minimal. The nonlinear regression parameters of an adsorption system are fitted by minimizing the errors. The lower error indicates the better performance of the predictive model. The statistical error functions can evaluate the fitting quality of the isotherm models.

In the current study, the mean absolute percentage error (MAPE), normalized average percentage error (NAPE), and chi-square (χ^2^) statistical methods are implemented to measure the errors.^12,54−56^ MAPE is the ratio of the difference between the experimental and predicted values to the predicted values of the experimental data findings. We defined a new error measurement parameter called normalized average percentage error (NAPE) as the ratio of the difference between the experimental and predicted values to the maximum value in the experimental data series. NAPE was introduced because the error in lower concentrations will be exaggerated, due to the low values in the denominator when using MAPE. NAPE calculates the error considering an isotherm’s maximum adsorption capacity (*Q*_max_), which will be more pragmatic and useful; chi-square is a statistical measurement to assess the goodness of fit between the experimental and predicted data points. It can be calculated as the sum of the squares of the differences between the experimental and predicted values divided by the predicted values. The errors are minimized between the experimental and predictive model values with the help of MS Excel Solver.^57,58^
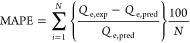
15
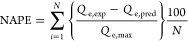
16
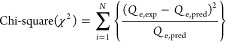
17where, *Q*_e,exp_ and *Q*_e, pred_ are the experimental and predicted values, respectively, *N* is the number of experimental data points, and *Q*_e,max_ is the maximum experimental value in the data.

## Results and Discussion

### Testing and Performance Evaluation of the JAMM Model Using Experimental Results

#### Study of Arsenic and Fluoride Adsorption Systems

To validate and assess the performance of JAMM model, we conducted a competitive adsorption experiment for arsenic(V) and fluoride systems as given below.

#### Materials and Methodology

The reagents disodium hydrogen arsenate (Na_2_HAsO_4_·7H_2_O) for As(V) and sodium fluoride (NaF) for F^–^ were used. Distilled water was employed to prepare the solutions during the experiments. The As(V) stock solution of 100 mg/L was prepared by dissolving 0.416 g of Na_2_HAsO_4_·7H_2_O in 1000 mL of distilled water, and the F^–^ stock solution of 100 mg/L was prepared by dissolving 0.213 g of NaF in 1000 mL. The commercially available activated carbon was chosen as an adsorbent. The insoluble and granular activated carbon was purchased from Darco, USA. The activated carbon particle size was 12–20 mesh, with the selectivity range of up to 57, and high-purity grades were obtained from Sigma-Aldrich.

The adsorption isotherm experiments were conducted in single and simultaneous systems by varying the initial concentrations from 0 to 100 mg/L. The volume of 50 mL of solution was taken in 250 mL conical flasks, and the pH of the solution was adjusted to 5 using NaOH or HCl. The activated carbon was dosed at 5 g/L. The experiments were conducted at 25 °C for 48 h with an agitation speed of 150 rpm. Subsequently, the solutions were filtered using microfilters. An atomic adsorption spectrometer was used to identify the arsenic concentration levels from the collected samples, and fluoride samples were measured with a Thermo Orion ion-selective fluoride meter.

**Figure 4:**
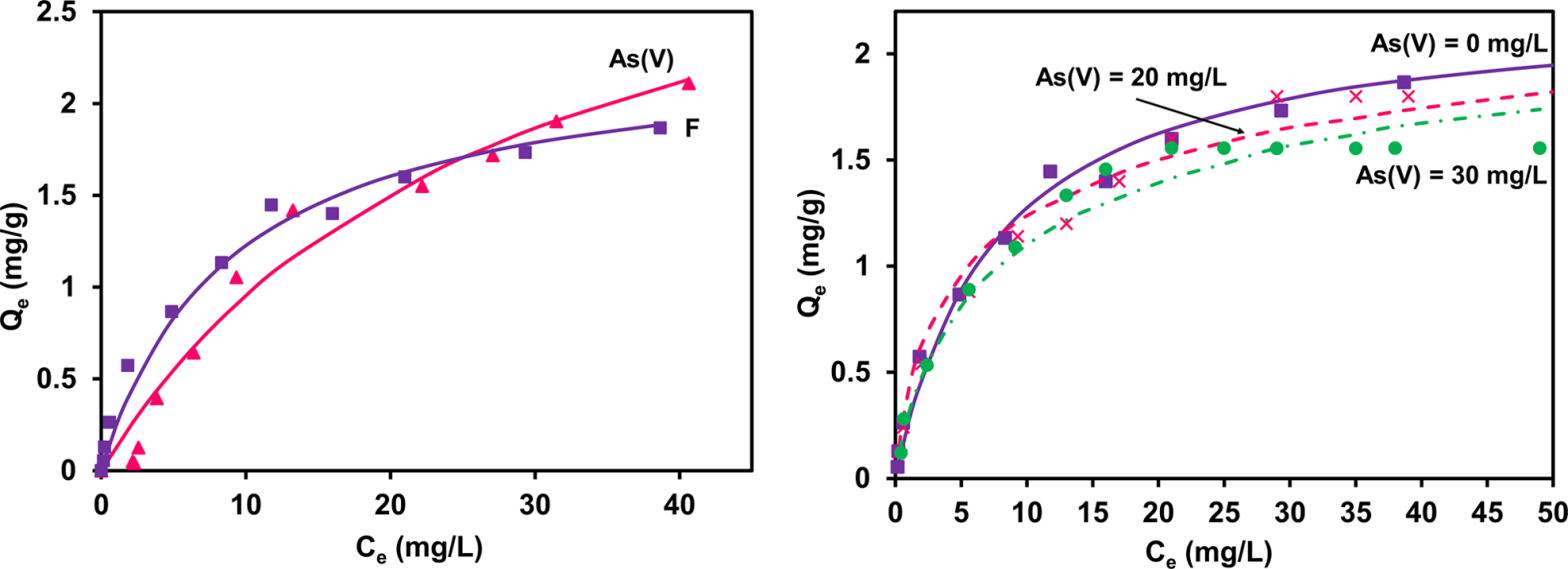
Langmuir isotherm model fit (solid lines) of single-component adsorption system of As(V) and F^–^ (left) and JAMM fit (dotted lines) of binary-component adsorption system at different initial concentrations of F and As(V) at 20 and 30 mg/L (right).

The experiments were conducted first for single-equilibrium As(V) and F^–^ adsorption, with activated carbon as an adsorbent. Adequate preliminary studies were conducted for optimal experimental adsorption conditions. This study focuses on the single and binary equilibrium data findings only, and not on kinetics. The fundamental nonlinearized Langmuir isotherm model (eq 1) was applied for the single-component adsorption system of As(V) and F^–^, separately, as indicated in Figure 4. The isotherm parameters *Q*_m_, the maximum adsorption capacity (mg/g), and *K*_L_ (L/mg), the Langmuir constant, were predicted by using MS Excel Solver by minimizing the average percentage errors. The correlation coefficient (*R*^2^) between the data and model was also estimated.

The binary equilibrium adsorption experiments of As(V) and F^–^ were investigated using activated carbon as an adsorbent, and the competitive adsorption behavior modeled using JAMM isotherm. The binary batch experiments were conducted at room temperature of 25 °C in an incubator shaker for 150 rpm placed for 48 h for equilibrium. However, the different initial concentrations of F^–^ ranging from 0 to 100 mg/L were used at a constant arsenic concentration of 20 and 30 mg/L. The activated carbon dosage was 5 g/L to the volume of 50 mL of solution. Several previous researchers have also studied competitive adsorption effects using similar procedure.^59,60^

#### Model Results

By employing the nonlinearized Langmuir isotherm model individually for the single-component adsorption system of As(V) and F^–^, the isotherm parameters *Q*_m_, the maximum adsorption capacity (mg/g), and *K*_L_, the Langmuir constant, were determined using MS Excel Solver by minimizing the average percentage errors (eqs 15 and 16), and the correlation coefficient (*R*^2^) was evaluated (Table 2). Furthermore, the isotherm parameters *Q*_m_ and *K*_L_ from single-component-predicted values were determined and utilized to simulate binary component adsorption systems.

Notably, the values of the maximum adsorption capacity of As(V) of *Q*_m,As(V)_ = 3.58 mg/g and the Langmuir constant of As(V) of *K*_L,As(V)_ = 0.036 L/mg were predicted by the excel solver. The maximum adsorption capacity of F of *Q*_m,F_ = 2.24 mg/g and the Langmuir constant of F^–^ of *K*_L,F_ = 0.13 L/mg, from the single-component system, were applied in the competitive JAMM (eq 14) isotherm for the simulation of the binary-component model. JAMM predicted the competitive isotherm parameters proficiently, as shown in Figure 4.

The assessed equilibrium JAMM isotherm parameters are listed in Table 2. Also, Table 3 represents the aqueous species’ molecular-level physical and electrostatic properties. Using these properties, we explore the reasons for the competitive adsorption behavior. We here introduce a new term, called the surface attractivity ratio, defined as the ratio of surface charge densities of two components. In the surface attractivity ratio of a system of A and B, if the surface attractivity ratio > 1, A will have preference over B. The calculated surface attractivity ratio of As(V) was 1.4, exceeding F^–^, as shown in Table 3, which describes that a small competition was offered by As(V). This further suggests that As(V) will be preferred over F^–^ due to its higher surface charge. The surface charge density of As(V) was higher, influencing the electrostatic interactions of As(V) with activated carbon and adsorbing more significantly than F^–^. The average percentage error (NAPE) was less than 5%, and the correlation coefficient (*R*^2^) was more than 0.99; close to 1 indicates a high performance, highlighting the JAMM isotherm model’s goodness of fit for As(V) and F^–^ in simultaneous removal.

**Table 2:** List of JAMM Isotherm Parameters for Single and Binary Adsorption System of As(V) and F^–^

no	isotherm	metal ion	parameters	*R*^2^	NAPE [%]	MAPE [%]	χ^2^
1	Langmuir	As(V)	*K*_L_ = 0.037 L/mg	0.98	3.93	21.90	0.56
			*Q*_m_ = 3.55 mg/g				
		F^–^	*K*_L_ = 0.13 L/mg	0.98	0.49	20.17	0.23
			*Q*_m_ = 2.24 mg/g				
2	JAMM	F^–^	*K*_L,*i*_ = 0.036 L/mg	0.99	4.69	8.67	0.09
			*K*_L,*j*_ = 0.13 L/mg	0.99	4.70	6.74	0.07
			*Q*_m_ = 2.24 mg/g				
			*n*_*i*_ = 0.20				
			*n*_*j*_ = 0.005				
			Φ_*i*_ = 1.85				
			*a*_*i*_ = 3.27				
			*a*_*j*_ = 0.45				
3	the average error of JAMM			0.99	4.7	7.77	0.08

**Table 3:** Atomic Level Properties of the Competing Species

no	properties	As(V)	F	Cd(II)	Zn(II)	Cu(II)	Cr(VI)	refs
1	species	HAsO_4_^2–^, H_3_AsO_4_, H_2_AsO_4_^–^	F^–^	Cd(OH)_2_, Cd(OH)_3_^–^, Cd(OH)^+^	Zn(OH)_2_, Zn(OH)_3_^–^, Zn(OH)_4_^–^, Zn(OH)^+^	Cu(OH)_2_, Cu(OH)_3_^–^, Cu(OH)_4_^2–^, Cu(OH)^+^	HCrO_4_^–^, H_2_CrO_4_, Cr_2_O_7_^2–^, CrO_4_^2–^	(61 and 62)
2	hydrated radii (Å)	>2–2.2	3.52	4.26	4.30	4.19	1.25	
3	ionic radii (Å)	0.58	1.19	0.97	0.74	0.73	0.52	
4	electronegativity (Pauling)	2.18	3.98	1.69	1.65	1.9	1.66	
5	surface area (SA), (Å^2^)	60.821	155.702	228.049	232.352	220.616	19.635	
6	surface charge density (Pauling/Å^2^)	0.0358	0.0256	0.0074	0.0071	0.0086	0.0845	
7	% surface charge density (Pauling/Å^2^)	3.58	2.56	0.74	0.71	0.86	8.45	
8	surface attractivity ratio	1.40	0.713	1.04	0.958	0.10	9.817	
9	competition	small relative competition		negligible relative competition		extreme relative competition		
10	inference	As(V) is a slightly stronger adsorbate compared to fluoride ion		both Cd and Cu(II) are similar in adsorption affinity		Cr(VI) is a very strong adsorbate compared to Cu(II)		

#### Statistical Model Performance Metrics

**Figure 5:**
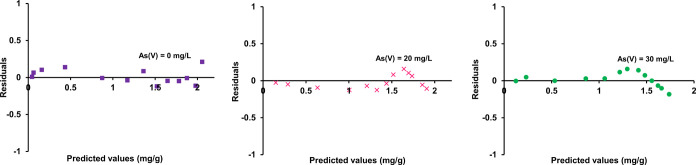
Analysis of the residual plot of As(V) and F^–^ of the JAMM model fit.

The correlation coefficient (*R*^2^) and other statistical error functions can determine the model quality assessment. The residual regression analysis is a critical tool for the interpretation of the fitting quality of the model. The residual plots are straightforward graphical plots indicating the predicted model’s acceptability or unacceptability. It is a scatterplot of randomly distributed data residuals determining the relevance of the designed model. Further, the positive residuals convey that the model prediction is low; negative values show that the model prediction is high; and residuals closer to the zero line indicate the accuracy of the predicted values. The distance between the residuals from the zero line displays the inaccuracy of the model fitting.^63−65^ Furthermore, the predicted values of the JAMM model closely align with the zero line, which indicates the accuracy of the isotherm model fitting, as in Figure 5.

**Figure 6:**
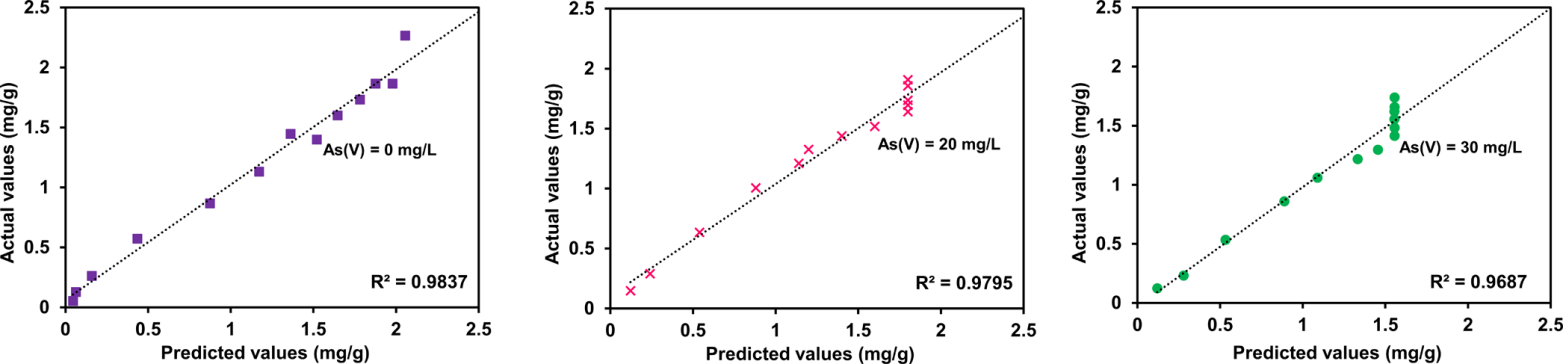
Relationship between the experimental and predicted values of As(V) and F^–^ of the JAMM model fit.

**Figure 7:**
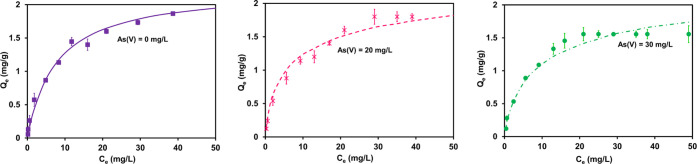
Analysis of error plots of As(V) and F^–^ of the JAMM model fit.

Additionally, as can be seen in Figure 6, the variation of data points of the measured and estimated values by the JAMM model was linear, with excellent *R*^2^ values, where most of the data points aligned to the straight line, and a slight deviation was observed in only in some experimental data. The JAMM model agreed well with the experimental predictions in the simultaneous adsorption system. Furthermore, the performance of the JAMM model was also validated by the standard deviation of the error plot. An error graph is a graphical representation associated with the uncertainty of each data point.^54,66^ The precision of the measured and estimated value deviation is statistically denoted in Figure 7.

### Case Study of Cadmium and Zinc Adsorption System

#### Materials and Methodology

**Figure 8:**
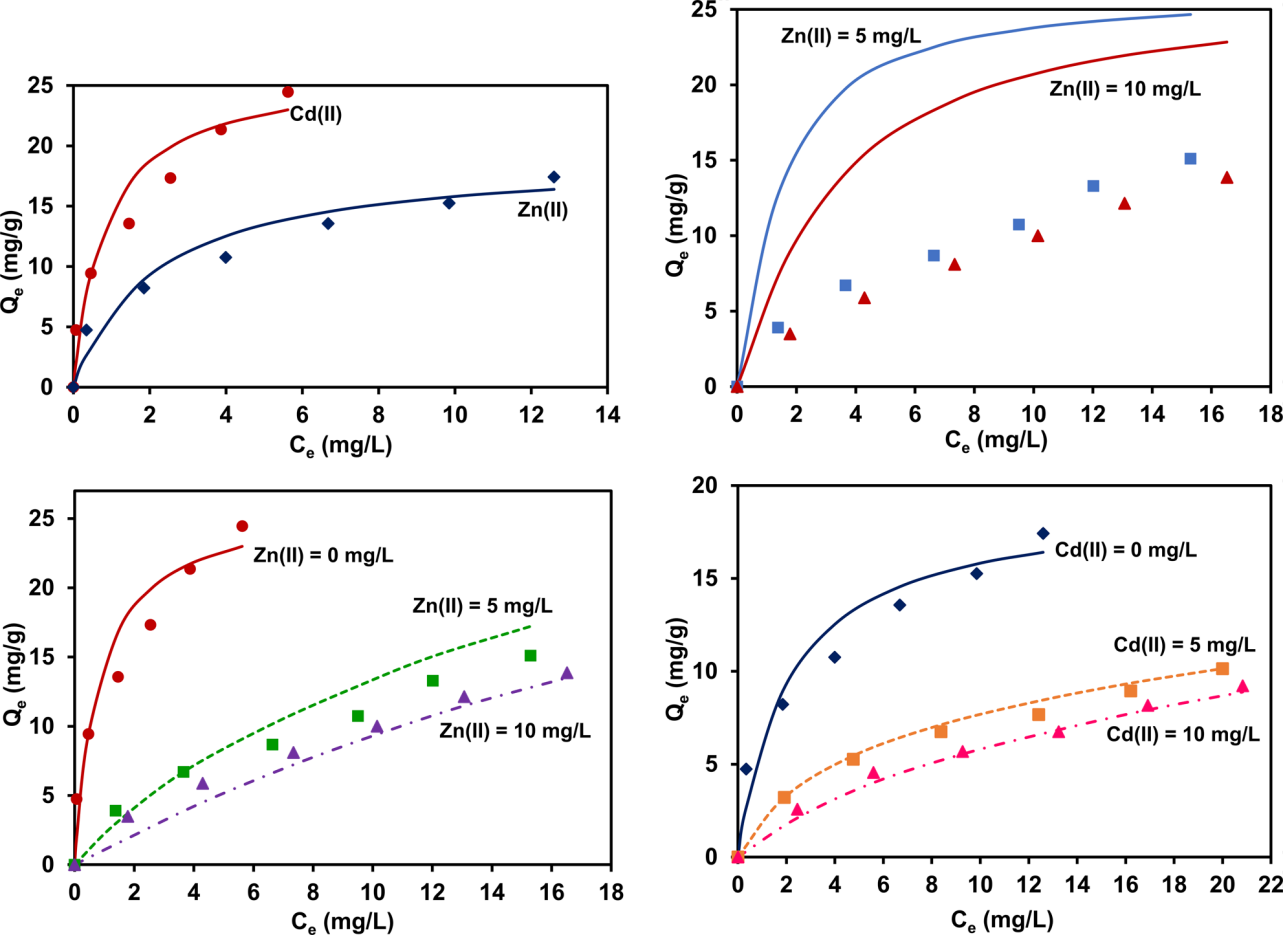
Langmuir isotherm model fit (solid lines) of the single-component adsorption system of Cd(II) and Zn(II) (left, top), JAMM fit (dotted lines) of binary-component adsorption system at different initial concentrations of Cd(II) and constant Zn(II) at 5 and 10 mg/L (left, bottom), EL isotherm fit of the binary-component adsorption system at different initial concentrations of Cd(II) and constant Zn(II) at 5 and 10 mg/L (right, top), and JAMM fit of the binary-component adsorption system at different initial concentrations of Zn(II) and constant Cd(II).

To validate and assess the JAMM model’s performance, a second adsorption case study from the literature involving Cd(II) and Zn(II) was considered. In the selected study,^12^ the single equilibrium adsorption of Cd(II) and Zn(II) was carried out using leonardite as an adsorbent. The batch experiments were conducted at 30 °C. The initial concentrations varied from 5 to 30 mg/L. The dosage of leonardite was 0.1 g in a volume of 100 mL of solution. A thermostatic water bath shaker was used at 120 rpm for 1 h. The extracted equilibrium data were sourced from the earlier research paper.^12^ The fundamental nonlinearized Langmuir isotherm model (eq 1) was employed on the single-component adsorption system of Cd(II) and Zn(II) separately, as shown in Figure 8.

The competitive batch experiments were conducted at 30 °C in a thermostatic water bath shaker at 120 rpm for 1 h. The different Cd(II) initial concentrations ranging from 5 to 30 mg/L were examined, with Zn(II) held constant at 5 and 10 mg/L. The binary solution was assessed with various ratios of initial concentrations of Cd(II) and Zn(II). The dosage of leonardite was 0.1 g taken to a volume of 100 mL of solution. The fundamental nonlinearized Langmuir isotherm model was applied for the single-component adsorption system of Cd(II) and Zn(II) individually. Isotherm parameters *Q*_m_, the maximum adsorption capacity (mg/g), and *K*_L_ (L/mg), the Langmuir constant, are predicted using MS Excel Solver by minimizing the average percentage errors. The correlation coefficient (*R*^2^) was also evaluated.

#### Model Results

The isotherm parameters *Q*_m_ and *K*_L_ from single-component adsorption were analyzed, and predicted values were utilized in JAMM to model the binary component systems. The binary data sets are also modeled using EL isotherm model (eq 2) for Cd(II) and Zn(II) to evaluate the EL model parameters by reducing the errors. The competitive data sets are next fitted simultaneously with JAMM isotherm by minimizing the errors between the experimental and predicted values, while also measuring the correlation coefficient (*R*^2^).

The results in Figure 8 (top right) indicate the failure of the extended Langmuir isotherm model in fitting the multicomponent system while using the estimated parameters of the single component adsorption system. The new computational approach isotherm model, JAMM, consequently mitigates this limitation (bottom left and bottom right) and gives good predictions. The JAMM isotherm model thus probably fixes the research gap of many models because it includes the mole fraction and the interaction coefficient between the adsorbates.

Table 4 shows the modeling parameters; the values of the maximum adsorption capacity of Cd(II) of *Q*_m,Cd(II)_ = 26.39 mg/g and the Langmuir constant of Cd(II) of *K*_L,Cd(II)_ = 1.2 L/mg were predicted. Also, the maximum adsorption capacity of Zn(II) of *Q*_m,Zn(II)_ = 19.16 mg/g and the Langmuir constant of Zn(II) of *K*_L,Zn(II)_ = 0.47 L/mg, achieved from a single-component analysis, were applied in the competitive isotherm data sets. JAMM (eq 14) isotherm simulates the binary-component model by lessening the errors simultaneously to all multicomponent data sets, as in Figure 8.

**Table 4:** JAMM Isotherm Parameters for the Single and Binary Systems of Cd(II) and Zn(II)

no	isotherm	metal ion	parameters	*R*^2^	NAPE [%]	MAPE [%]	χ^2^
1	Langmuir	Cd(II)	*K*_L_ = 1.2 L/mg	0.90	7.09	29.83	1.05
			*Q*_m_ = 26.39 mg/g				
		Zn(II)	*K*_L_ = 0.47 L/mg	0.83	6.78	19.56	0.45
			*Q*_m_ = 19.16 mg/g				
2	JAMM	Cd(II)	*K*_L,*i*_ = 1.2 L/mg	0.82	9.31	14.49	1.38
			*K*_L,*j*_ = 0.47 L/mg	0.76	6.61	23.62	1.96
			*Q*_m_ = 26.39 mg/g				
			*n*_*i*_ = 0.51				
			*n*_*j*_ = 0.41				
			Φ_*i*_ = 2.99				
			*a*_*i*_ = 0.01				
			*a*_*j*_ = 0.01				
		Zn(II)	*K*_L,*i*_ = 1.2 L/mg	0.97	2.83	3.66	0.11
			*K*_L,*j*_ = 0.47 L/mg	0.98	3.34	7.84	0.10
			*Q*_m_ = 19.16 mg/g				
			*n*_*i*_ = 0.49				
			*n*_*j*_ = 0.41				
			Φ_*j*_ = 2.12				
			*a*_*i*_ = 0.01				
			*a*_*j*_ = 0.01				
3	the average error of JAMM			0.88	5.5	12.4	0.88

Similar research studies were conducted on Cd(II) and Zn(II) earlier.^67^Table 4 gives the JAMM predictions and evaluations of the competitive isotherm parameters. Also, Table 3 displays the calculated surface attractivity ratio of 1.04, which clarifies a negligible competition between Cd(II) and Zn(II), implying that both Cd(II) and Zn(II) adsorb at a similar affinity as it was close to unity. Additionally, the similarity in the surface charge density of Cd(II) and Zn(II) exhibits nearly identical electrostatic interactions with leonardite. The average percentage error (NAPE) was less than 3% in the case of Zn(II) and less than 8% in the case of Cd(II). The average NAPE value was around 5% for the two adsorbates. The correlation coefficient (*R*^2^) was near 0.9, demonstrating the model’s accurate fit for Cd(II) and Zn(II) in the simultaneous adsorption, as seen in Figure 8.

### Case Study of the Copper and Chromium Binary Adsorption System

#### Materials and Methodology

**Figure 9:**
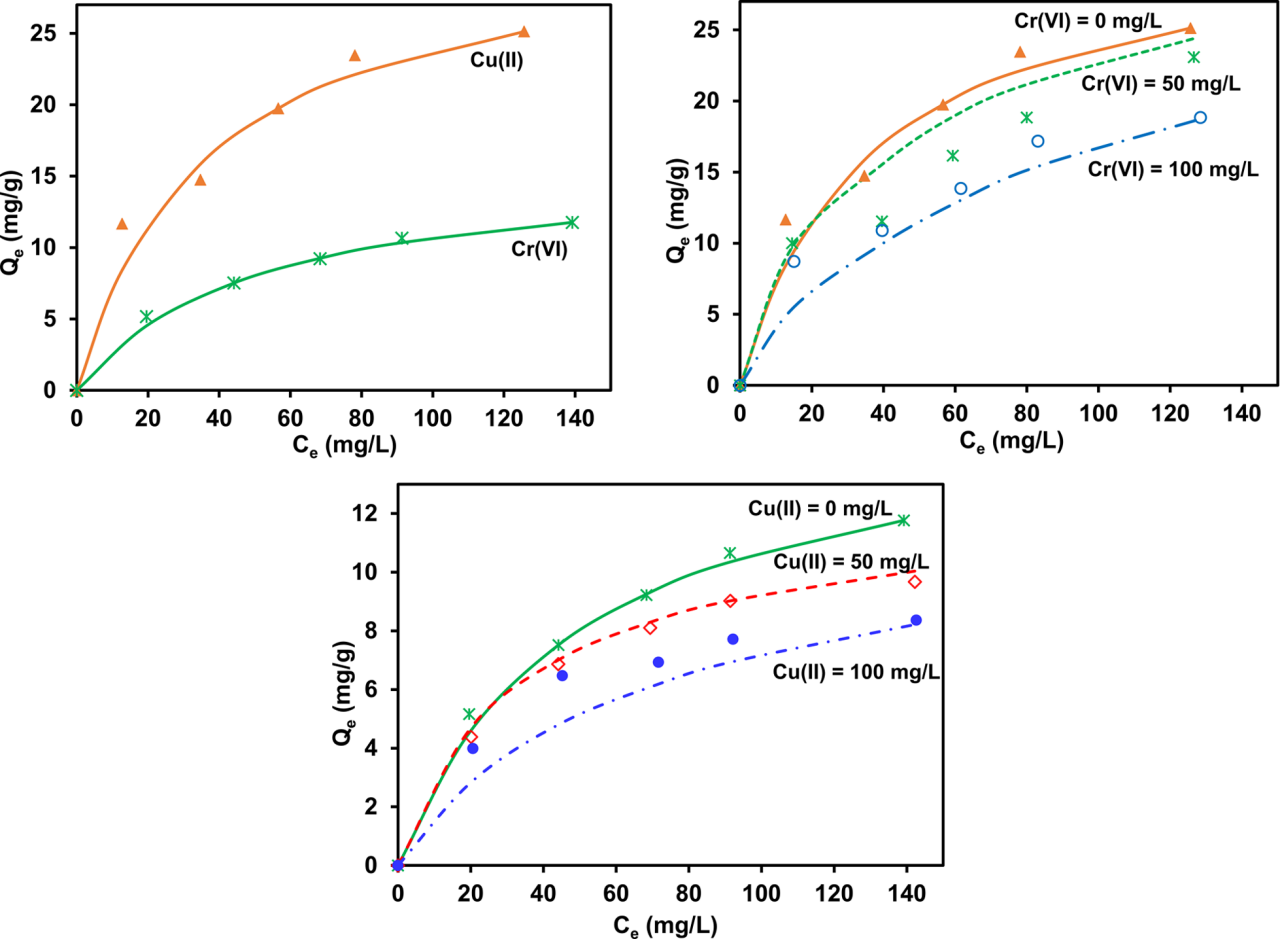
Langmuir isotherm model fit (solid lines) of the single-component adsorption system of Cu(II) and Cr(VI) (left, top), JAMM fit (dotted lines) of the simultaneous adsorption system at different initial concentrations of Cu(II) and Cr(VI) at 50 and 100 mg/L (right, top), JAMM fit of simultaneous adsorption system at different initial concentrations of Cr(VI) and Cu(II) at 50 and 100 mg/L (bottom).

Another case study of equilibrium adsorption of Cu(II) and Cr(VI) using dried *Chlorella vulgaris* as an adsorbent^68^ was investigated in this study for the validation of the JAMM isotherm. The batch experiments were conducted at 25 °C. The initial concentrations varied from 25 to 250 mg/L. The dosage of alga was 1 g/L for a volume of 100 mL of solution. The flasks were agitated using a shaker for 24 h. We extracted and examined the equilibrium data from the paper,^68^ and the nonlinearized Langmuir isotherm model (eq 1) was employed on the single-component adsorption system of Cu(II) and Cr(VI) separately, as displayed in Figure 9. The isotherm parameters *Q*_m_, the maximum adsorption capacity (mg/g), and *K*_L_ (L/mg), the Langmuir constant, are fitted using MS Excel Solver by minimizing the average percentage errors, and the correlation coefficient (*R*^2^) was also evaluated.

The binary data were taken from the abovementioned previous research study.^68^ The simultaneous equilibrium adsorption experiments of Cu(II) and Cr(VI) were carried out by using dried *C. vulgaris* as an adsorbent. The competitive batch experiments were conducted at 25 °C, by agitating in a shaker for 24 h. The different Cu(II) input concentrations ranging from 25 to 250 mg/L were used, with Cr(VI) being held constant at 50 and 100 mg/L as shown in above figure. The binary solution was prepared with various initial Cu(II) and Cr(VI) concentrations. The dosage of alga was 1 g/L.

#### Model Results

The Langmuir isotherm model (eq 1) was applied (top left) to the single-component adsorption system of Cu(II) and Cr(VI) independently. Isotherm parameters *Q*_m_, the maximum adsorption capacity (mg/g), and *K*_L_ (L/mg), the Langmuir constant, are predicted using MS Excel Solver by minimizing the average percentage errors. Thereafter, the isotherm parameters *Q*_m_ and *K*_L_ from single-component adsorption were utilized to model competitive systems using the JAMM equation. The competitive data sets were simultaneously fitted with JAMM by minimizing the errors between experimental and predicted values using Excel Solver. The chi-square, NAPE, MAPE, and correlation coefficient (*R*^2^) values were also evaluated for the model predictions.

Table 5 shows the parameter results from the modeling and simulation; the values of the maximum adsorption capacity of Cu(II) of *Q*_m,Cu(II)_ = 32.38 mg/g and the Langmuir constant of Cu(II) of *K*_L,Cu(II)_ = 0.028 L/mg were predicted. Also, the maximum adsorption capacity of Cr(VI) of *Q*_m,Cr(VI)_ = 15.96 mg/g and the Langmuir constant of Cr(VI) of *K*_L,Cr(VI)_ = 0.02 L/mg, obtained from the mono-component adsorption system, were applied in the competitive isotherm data sets. The JAMM (eq 14) isotherm simulated the binary component model by lessening the errors simultaneously in all multicomponent data sets, as shown in Figure 9.

**Table 5:** JAMM Isotherm Parameters for Single and Binary Systems of Cu(II) and Cr(VI)

no	isotherm	metal ion	parameters	*R*^2^	NAPE [%]	MAPE [%]	χ^2^
1	Langmuir	Cu(II)	*K*_L_ = 0.028 L/mg	0.90	4.53	10.31	1.41
			*Q*_m_ = 32.38 mg/g				
		Cr(VI)	*K*_L_ = 0.020 L/mg	0.98	1.68	3.53	0.10
			*Q*_m_ = 15.96 mg/g				
2	JAMM	Cu(II)	*K*_L,*i*_ = 0.028 L/mg	0.73	9.24	11.93	1.76
			*K*_L,*j*_ = 0.020 L/mg	0.79	7.34	17.41	2.25
			*Q*_m_ = 32.38 mg/g				
			*n*_*i*_ = 0.01				
			*n*_*j*_ = 0.01				
			Φ_*i*_ = 3.16				
			*a*_*i*_ = 0.89				
			*a*_*j*_ = 1.02				
		Cr(VI)	*K*_L,*i*_ = 0.028 L/mg	0.98	2.07	2.91	0.04
			*K*_L,*j*_ = 0.020 L/mg	0.60	10.36	19.04	1.11
			*Q*_m_ = 15.96 mg/g				
			*n*_*i*_ = 0.01				
			*n*_*j*_ = 0.01				
			Φ_*j*_ = 2.38				
			*a*_*i*_ = 0.89				
			*a*_*j*_ = 1.02				
3	the average error of JAMM			0.78	7.25	12.82	1.29

The evaluated equilibrium JAMM isotherm parameters are listed in Table 5. The calculated surface attractivity ratio of Cu(II) was 0.102, as shown in Table 3, which suggests that a comparatively tenacious competition was provided by Cr(VI), and that Cr(VI) was a strong adsorbate compared to Cu(II). Furthermore, the surface charge density of Cr(VI) exceeds that of Cu(II), which elucidates that the electrostatic interactions with the dried *C. vulgaris* adsorbent were higher with Cr(VI). However, the interaction coefficient of Cu(II) was greater than Cr(VI) in competitive system, which suggests that *C. vulgaris* prefers to adsorbs more Cu(II) than Cr(VI), relative to their single component adsorption capacity. The average NAPE was around 7%, and the correlation coefficient (*R*^2^) was around 0.8, suggesting a slightly less satisfactory fit than the previous case studies. This could also be due to more experimental errors in the Cu(II) and Cr(VI) systems in the simultaneous removal or reduced model effectiveness for this system. Many times, experimental errors are around 5–10% are routinely found due to instrumental inaccuracies.

## Discussion

In combination, several pollutants such as arsenic, fluoride, copper, chromium, cadmium, and zinc adversely affect the quality of groundwater and water resources. The co-occurrence of these pollutants in water is toxic and causes many public health concerns, possibly in a synergistic manner. The multicomponent adsorption treatment process offers the unique advantage of removing multiple pollutants in the aqueous phase at the same time. Currently however multicomponent modeling methods are inadequate. For example, the extended Langmuir (EL) model was unable to predict multicomponent adsorption using single component data (Figure 8). Surface complexation modeling method can be used for modeling such multicomponent systems, but is a complex and time-intensive framework compared with simpler adsorption isotherms. Some critical aspects of multicomponent adsorption are preference, competitive nature, interaction between the components, affinity toward the adsorbents, surface area of the adsorbent, and selectivity of pollutants toward the adsorbent of the adsorption system. Specific factors influencing the adsorption system are overlooked in the traditional multicomponent isotherm models. These omissions are rectified and incorporated in the JAMM model. The JAMM isotherm model is more straightforward, systematic, and clear for analyzing multicomponent adsorption systems. This analytical, computational multicomponent isotherm framework considers substantial terms such as the interactions between pollutants and adsorbents, mole fractions, affinity factor, and heterogeneity index, thereby providing more nuanced adsorption mechanisms.

To validate the JAMM model’s performance, experiments pertaining to As(V) and F^–^ on activated carbon were conducted by us. From our analysis, the affinity factor of As(V) was found to be more than that of F^–^. The heterogeneity index lies below 1, showing that the surface was heterogeneous. The interaction coefficient shows that As(V) interacted more with the adsorbent surface than F^–^. The activated carbon surface carries a positive charge at pH 5, and since both As(V) and F^–^ are anions when dissolved in water, a positively charged surface promotes enhanced adsorption capacity. The maximum adsorption capacity was higher for As(V) than for F^–^. Therefore, the As(V) mole fraction strongly influences the simultaneous removal of the adsorption system. Furthermore, the molecular parameters, including ionic radii, hydrated radii, electronegativity, ion diffusivity, surface charge density, and surface attractivity ratio, affect the competitive interaction mechanism between As(V) and F^–^. The ionic radii of As(V) (0.58 Å) and F^–^ (1.19 Å); As(V) enable greater accessibility to the active sites of activated carbon. The smaller the ionic radii, the more is the adsorption.

The difference in the adsorption behavior between As(V) and F^–^ can also be attributed to the smaller molecular size of As(V), which can penetrate deeper into the adsorbent, causing steric hindrance and pore restriction for F^–^. On examining the hydrated radius of As(V) (2.2 Å), which is smaller than that of F^–^ (3.52 Å), the size difference suggests that As(V) was more effective in adsorption due to its reduced hydrated radius. The smaller the size, the more is the ion diffusivity and mobility of As(V), reaching the active sites faster than that of bulkier F-ions. Moreover, the higher % surface charge density of As(V) (3.58) compared to F^–^ (2.56) leads to the selective As(V) adsorption on activated carbon in a competitive system. This disparity in electronegativity favors the preferential adsorption of As(V) over F^–^, depending on the presence of species in the aqueous phase. The surface attractivity ratio of As(V) (1.4) was more significant than that of F^–^, describing a relatively small competition by As(V). Additionally, the surface charge density of As(V) was higher, which influenced the electrostatic interactions of As(V) with activated carbon, and hence, As(V) adsorbed more than F^–^ (see Table 3).

The coefficient of determination (*R*^2^) of As(V) and F^–^ consistently exceeded 0.98, and minimum χ^2^ errors, implying the accuracy and precision of the model fit and attesting to the model’s robustness and reliability, statistically. Also, the average percentage error remained below 5% for As(V) and F^–^, proving the adequacy and credibility of the analytical framework JAMM isotherm (see Figure 4). The statistical error analysis, the standard deviation error plots, and the residual regression analysis were conducted to interpret the fitting quality of As(V) and F^–^; residual graphical plots and error plots indicated the model’s acceptability. The scattered plot of randomly distributed data residuals suggested the relevance of the JAMM model. The residuals were closer to the zero line, indicating the multicomponent system’s accuracy of predicted values of As(V) and F^–^ (see Figure 5). The deviation of measured data points and estimated values by the JAMM model was linear with a better *R*^2^ value; the JAMM model agreed well with the experimental predictions in the simultaneous system of As(V) and F^–^ (see Figure 6). Figure 7 gives the error plots, where the experimental variation with predicted values was assured.

Similarly, a second case study was considered to validate the model performance of the Cd(II) and Zn(II) adsorption systems. On comparing the modeled systems of Cd(II) and Zn(II), the interaction coefficient of Cd(II) was found to be higher than that of Zn(II), indicating that the influence of the interaction selectivity was significant on the adsorption of Cd(II) than Zn(II). Therefore, the effect of the mole fraction of Cd(II) was more predominant than Zn(II). Correspondingly, the surface charge density of Zn(II) was equivalent to the surface charge density of Cd(II), which indicates the fact that the affinity of Cd(II) and Zn(II) toward the adsorbent was similar. There was negligible relative competition. Both components were almost equally strong. The surface was heterogeneous, as the heterogeneity index lies below 1 (see Table 4). The ionic radius of Cd(II) was 0.97 Å and that of Zn(II) was 0.74 Å. The greater ionic radii result in lower accessibility to the active sites and can slightly reduce the adsorption process.

The hydrated radius of Cd(II) (4.26 Å), which was slightly similar to that of Zn(II) (4.30 Å), may not differ from each other. Moreover, the electronegativity of Cd(II) (1.69) compared to that of Zn(II) (1.65) was almost identical, and the adsorption slightly changed in a simultaneous system. Depending on the Cd(II) and Zn(II) species present, the higher the electronegativity, the higher is the adsorption. The surface attractivity ratio of Cd(II) (1.04) showed negligible competition between Cd(II) and Zn(II), implying that both Cd(II) and Zn(II) adsorb at similar affinity. Additionally, the similarity in the surface charge density of Cd(II) and Zn(II) exhibits nearly identical electrostatic interactions with leonardite (Table 3). The coefficient of determination (*R*^2^) of Cd(II) and Zn(II) was consistently high, implying that the model fits and attests to the model’s accuracy. Also, the average percentage errors remained below 10%, and the minimum χ^2^ errors further established the superiority of JAMM’s predictions (see Figure 8).

Additionally, the JAMM isotherm modeling framework was confirmed using another case study of Cu(II) and Cr(VI) simultaneous adsorption system. The simulation results are as in Table 5. The Cu(II) and Cr(VI) simultaneous adsorption systems gave the interaction coefficient of Cu(II) as higher (3.16) than that of Cr(VI) at (2.38), denoting that Cu(II) adsorption impacts more on the adsorption sites, which suggests that *C. vulgaris* prefers more Cu(II) than Cr(VI) than stoichiometrically predicted. Also, the maximum single component uptake of Cu(II) was more significant than Cr(VI). The heterogeneity index lies below 1, suggesting that the surface was heterogeneous. The ionic radius of Cu(II) is 0.73 Å and that of Cr(VI) is 0.52 Å; Cr(VI) enables greater accessibility to the active sites of the adsorbent. The difference in the adsorption behavior between Cu(II) and Cr(VI) can also be attributed to the smaller molecular size of Cr(VI), which can penetrate deeper into the adsorbent, causing steric hindrance and pore restriction for Cu(II).

On comparing the hydrated radius of Cu(II) (4.19 Å), which is greater than that of Cr(VI) (1.25 Å), the difference in size suggests that Cu(II) was less effective in adsorption due to its high hydrated radius. However, the higher electronegativity of Cu(II) (1.9) compared with Cr(VI) (1.66) suggests the selectivity of Cu(II) adsorption in a binary system. The effect of electronegativity depends on the species, favoring the adsorption of Cu(II) over Cr(VI). The surface attractivity ratio of Cu(II) (0.102) clarifies the comparatively extreme competition by Cr(VI). Furthermore, the % surface charge density of Cr(VI) of 8.45 units elucidates better electrostatic interactions with the dried *C. vulgaris* adsorbent than that of Cu(II) which was 0.86 units (see Table 3). JAMM isotherm was able to simulate the adsorption system well. The correlation coefficient (*R*^2^) of Cu(II) and Cr(VI) was moderately good, and the NAPE remained >7%. The average *R*^2^ value was 0.79, showing a good model fit (see Figure 9).

The average errors of the three case studies are summarized in Table 6. The overall average *R*^2^ value was 0.86, and the average χ^2^ value was 0.69. The average NAPE was 6.05%, and the average MAPE was 11.63%. Thus, the error analysis showed a good fit by the JAMM model for the experimental data.

**Table 6:** Summarized Average Error Parameters of the Simulated Multicomponent Systems

no	adsorption systems	*R*^2^	NAPE (%)	MAPE (%)	χ^2^
case study-I (our experimental study)	fluoride	0.99	4.7	7.7	0.08
case study-II (literature)	cadmium	0.79	7.96	19.06	1.67
	zinc	0.97	3.09	5.75	0.11
case study-III (literature)	copper	0.76	8.30	14.67	2
	chromium	0.79	6.22	10.98	0.58
the average errors of all the case studies		0.86	6.05	11.63	0.69

In conclusion, the interaction coefficient and mole fraction eminently contribute to the competitive system’s adsorption behavior. JAMM’s exceptional predictive capabilities have demonstrated its efficacy in correctly predicting the adsorption parameters and comprehending the complex behaviors that appear in multicomponent systems thorough validation and simulation of diverse case studies.

Hence, JAMM might provide exciting opportunities in chemical engineering, environmental science, and material science applications by coming through as a potent tool for analyzing and forecasting a multicomponent adsorption behavior. It enables one to delve deeper into complex competitive adsorption mechanisms and use the acquired knowledge to make prudent choices for many practical adsorption applications. The JAMM isotherm can contribute substantially to exploring the competition adsorption behavior, molecular level interactions, and surface behavior, which provides more thorough understanding and accurate multicomponent adsorption predictions. Overall, the JAMM isotherm advances the comprehension of multicomponent adsorption phenomena and can accelerate development in multiple industries and scientific fields. Its integration into contemporary research and technology might help further improve our insights to multicomponent adsorption in various domains.

## Conclusions

A new multicomponent model (JAMM) was developed and applied to simulate various competitive adsorption systems. The new analytical approach comprehensively provided a reliable model for multicomponent adsorption systems. The JAMM isotherm allayed the limitations of existing multicomponent isotherm models. The JAMM isotherm incorporates reasonable descriptions of the interaction mechanism, affinity, heterogeneity, and mole fraction of pollutants. The JAMM isotherm was developed using our experimental competitive system of As(V) and F^–^ on activated carbon, which fitted well with low errors (average NAPE 4.7%) and attained a comparatively good fit of *R*^2^ (0.99) . The detailed error plots, residual regression analysis, and standard deviation errors further outlined the exceptional quality of the JAMM model fitting. The JAMM isotherm was then validated using binary equilibrium adsorption of Cd(II) and Zn(II), which also yielded excellent fitting parameters with the average NAPE of Cd(II), 7.96%, and Zn(II) as 3.09%. Additionally, we further tested the model using a Cu(II) and Cr(VI) competitive system, where the JAMM isotherm again showed robust predictions with an average NAPE of Cu(II) of 8.3% and Cr(VI) as 6.22%. Thus the JAMM isotherm was tested with three experimental case studies, with negligible, small, and high relative competition systems of adsorbates, and excellent predictions were obtained in all the adsorption systems. The overall average *R*^2^ value was 0.86 for all the three case studies, and the chi-square error was 0.69. The overall average MAPE was 11.63%, and average NAPE was 6.05%. The validation studies therefore suggest that the JAMM isotherm model can be implemented for competitive adsorption systems and constitutes an improved multicomponent isotherm approach. In summary, the JAMM isotherm model proves to be a promising approach for exploring multicomponent adsorption systems and provides an avenue for multicomponent adsorption modeling, especially for aqueous systems.
